# Randomized evaluation of an online single-session intervention for minority stress in LGBTQ+ adolescents

**DOI:** 10.1016/j.invent.2023.100633

**Published:** 2023-06-07

**Authors:** J. Shen, A. Rubin, K. Cohen, E.A. Hart, J. Sung, R. McDanal, C. Roulston, I. Sotomayor, K.R. Fox, J.L. Schleider

**Affiliations:** a Department of Psychology, Stony Brook University, United States of America; b Department of Psychology, University of Denver, United States of America; c Department of Medical Social Sciences, Feinberg School of Medicine, Northwestern University, United States of America

**Keywords:** Single-session intervention, Minority stress, Adolescents, LGBTQ+, Sexual minorities, Gender minorities

## Abstract

**Background:**

LGBTQ+ youth face myriad adverse health outcomes due to minority stress, creating a need for accessible, mechanism-targeted interventions to mitigate these minority stress-related risk factors. We tested the effectiveness and acceptability of Project RISE, an online single-session intervention designed to ameliorate internalized stigma and improve other outcomes among LGBTQ+ youth. We hypothesized that youth assigned to RISE (versus a control) would report significantly reduced internalized stigma and increased identity pride at post-intervention and at two-week follow-up and would find RISE acceptable.

**Methods:**

We recruited adolescents nationally through Instagram advertisements in May 2022 (*N* = 538; *M* age = 15.06, *SD* age = 0.97). Participants were randomly assigned to RISE or an information-only control and completed questionnaires pre-intervention, immediately post-intervention, and two weeks post-intervention. Inclusion criteria included endorsing: (1) LGBTQ+ identity, (2) age 13–16, (3) English fluency (4) Internet access, and (5) subjective negative impact of LGBTQ+ stigma.

**Results:**

Relative to participants in the control condition, participants who completed RISE reported significant decreases in internalized stigma (*d* = −0.49) and increases in identity pride (*d* = 0.25) from pre- to immediately post-intervention, along with decreased internalized stigma (*d* = −0.26) from baseline to two-week follow-up. Participants rated both RISE and the information-only control as highly, equivalently acceptable.

**Conclusions:**

RISE appears to be an acceptable and useful online SSI for LGBTQ+ adolescents, with potential to reduce internalized stigma in both the short- and longer-term. Future directions include evaluating effects of Project RISE over longer follow-ups and in conjunction with other mental health supports.

## Highlights

• Minority stress exacerbates internalized stigma in LGBTQ+ adolescents.
• Project RISE is an online single-session program designed to reduce internalized stigma.
• Relative to a control, Project RISE reduced internalized stigma in LGBTQ+ youth.
• Project RISE also led to short-term improvements in hopelessness and self-hate.
• Project RISE appears to be an acceptable, useful online support for LGBTQ+ youth.

## 1 Introduction

LGBTQ+ youth routinely face discrimination and stigma related to LGBTQ+ identity (e.g., violence, victimization), which increases their risk for experiencing mental health problems compared to their cisgender, heterosexual peers (Chodzen et al., 2019; Guz et al., 2021; Marshal et al., 2011; Plöderl and Tremblay, 2015). Minority stress theory (MST) serves as a framework for understanding how cisheterosexism-related experiences exacerbate these problems, such as depression, hopelessness, and self-hate, over time (Brooks, 1981; Chodzen et al., 2019; Fulginiti et al., 2021; Meyer, 1995; Nappa et al., 2022). MST posits several pathways through which chronic, identity-based stressors undermine LGBTQ+ health, including discrimination and stigma because of one's identity, *internalization* of stigmatizing attitudes towards one's own identity (i.e., internalized stigma), *expectations of interpersonal rejection* due to one's identity, and LGBTQ+ identity concealment (Hatzenbuehler, 2009). Growing literature has also suggested identity pride as a pathway to resilience in the face of minority stress, and the importance of increasing pride (Delozier et al., 2020).

### 1.1 Potential targets of intervention in minority stress theory

Although structural or systemic interventions (e.g., via legislation or policies) are needed to address the myriad mechanisms by which minority stress can undermine LGBTQ+ health, there are other mechanisms, including intrapersonal factors, which may be targeted via individual-level support. For instance, intrapersonal stressors such as internalized stigma may be more immediately modifiable than those related to structural or interpersonal levels, such as anti-LGBTQ+ policies or cultural attitudes. At the intrapersonal level, interactive interventions beyond didactic educational interventions remain largely understudied (Chaudoir et al., 2017; Mandel, 2014; Proujansky and Pachankis, 2014). Internalized stigma has been minimally addressed in evidence-based therapies for LGBTQ+ youth, with most LGBTQ+-tailored interventions being designed for sexual minority adult men and young adults (Israel et al., 2019; Layland et al., 2020; Lin and Israel, 2012; Pachankis et al., 2020). As such, it remains critical to gauge whether targeting modifiable intrapersonal factors, such as internalized stigma, can help improve mental health and well-being for LGBTQ+ youth.

### 1.2 Accessibility of interventions and single-session interventions

There has been a longstanding dearth of accessible, evidence-based mental health supports for LGBTQ+ youth (Salerno et al., 2020). In addition to traditional barriers to healthcare (e.g., logistical, financial), LGBTQ+ youth experience additional barriers (e.g., familial rejection, invalidation; Roe, 2017) as well as a lack of affirming care options (Salerno et al., 2020). Affirming, evidence-based mental health supports for LGBTQ+ youth must be made broadly, easily accessible.

Digital single-session interventions (SSIs) are well-positioned to provide scalable, low-cost, effective tools for improving short-term risk factors for adverse health outcomes such as hopelessness, perceived control, and agency, in addition to longer-term well-being, as evidenced through reductions in symptoms related to depression, anxiety, and trauma (Schleider and Weisz, 2018). SSIs targeting effective coping strategies have demonstrated acceptability and effectiveness in LGBTQ+ adolescents nationwide (Schleider et al., 2022). Additionally, online SSIs are particularly well-suited to increase access to affirming, evidence-based care for LGBTQ+ youth, as they are self-guided, not location-dependent, relatively brief (e.g., 15–25 min), and eliminate premature treatment dropout problems. Previous research suggests that these SSIs are equally acceptable and helpful for LGBTQ+ and cisgender/heterosexual youth (McDanal et al., 2022). Building on existing online SSIs for LGBTQ+ adults which address factors related to understanding and coping with minority stress and its sequelae (e.g., Israel et al., 2019; Israel et al., 2021a; Israel et al., 2021b), the current study aimed to establish the efficacy of an online SSI designed specifically for LGBTQ+ youth.

### 1.3 The current study

We tested the immediate and 2-week effects of a novel online, self-guided SSI, ‘Project RISE.’ Drawing from best-practices in affirming treatment for LGBTQ+ populations (Pachankis et al., 2022), SSI design (Schleider et al., 2022), and co-creation models of intervention development, we designed Project RISE to systematically reduce internalized stigma, an intraindividual, and potentially modifiable, facet of minority stress.

We hypothesized that, compared to participants assigned to a minority stress information-only control condition, participants assigned to RISE would show: significantly reduced internalized stigma (co-primary outcome), increased identity pride (co-primary outcome), and hopelessness (secondary outcome) both immediately post-intervention and at two-week follow-up; as well as reduced self-hate at post-intervention and reduced depression symptoms at two-week follow-up (both secondary outcomes). We also hypothesized that participants assigned to RISE would rate the intervention as acceptable based on established user-feedback metrics for online SSIs.

## 2 Method

**Fig. 1:**
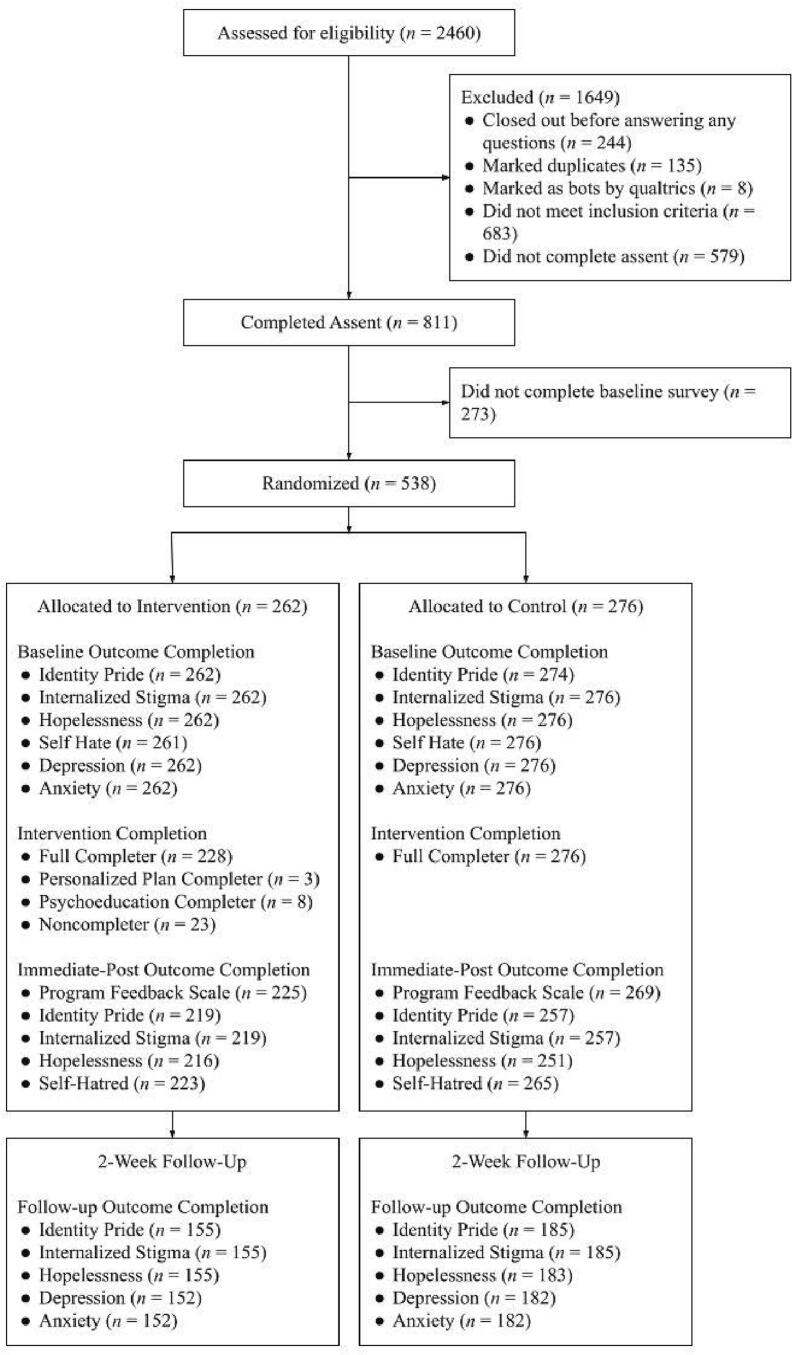
CONSORT diagram.

Study procedures were approved by the University of Denver Institutional Review Board (IRB) and were pre-registered on Open Science Framework (OSF; https://osf.io/es3zb).^3^ Participants were recruited online through advertisements on social media (i.e., Instagram; see Supplementary Fig. 1) within a one-week period in May 2022. Inclusion criteria included: (1) LGBTQ+ identity, (2) Age 13–16, (3) English fluency, (4) consistent Internet access, and (5) positive endorsement (i.e., ≥1 on a zero to ten scale) to the question, “Has LGBTQ+ stigma had a negative impact on your life?” Individuals who did not meet inclusion criteria, who exited the study prior to condition randomization, or who did not meet quality-check criteria (i.e., participants marked as providing duplicate responses per IP address, marked at bots by Qualtrics' internal bot-detection software, and who failed an attention-check item in the screening survey; see CONSORT diagram in Fig. 1) were excluded from analysis. Given the minimal risk posed by this study, as well as the barriers associated with requiring parental permission (particularly for LGBTQ+ minors) to participate in research, a waiver of parental permission was obtained from the University of Denver IRB.

At baseline (after completion of pre-intervention questionnaires), each participant was randomly assigned to RISE or to an information-only control condition. Participants also completed questionnaires immediately post-intervention and two weeks later in an optional follow-up assessment. Participants earned $10 for completing each of two assessment surveys.

Project RISE is a 20–30 min self-guided SSI explicitly targeting internalized stigma and minority stress reactions. RISE includes five general content sections: (1) an introduction to minority stress, privilege, and marginalization; (2) psychoeducation on the effects of minority stress; (3) stories from other youths about their experiences with minority stress; (4) interactive components wherein participants reflect on their identities and experiences with minority stress, identify related emotions and cognitions, and determine actionable, values-based needs; and (5) an exercise in which participants identify a coping statement to help them get through minority stress. Finally, participants receive an action card including their coping statement; their emotions, cognitions, and needs when minority stress arises; and strategies to act on their needs (see Supplementary Fig. 2). The SSI also includes an optional exercise where youths can share advice based on what they learned from the SSI. The full intervention is viewable here: https://osf.io/ktcd9.

In the information-only control condition, participants received an illustrated document discussing the concept of minority stress. The document included an age-appropriate explanation of MST, open-ended questions to guide participants in reflecting on how the theory applies to their own lives, and links to additional online educational resources on minority stress. Regardless of condition, participants were provided with a comprehensive resource guide, and participants assigned to the control condition were informed that they would receive delayed access to RISE.

### 2.1 Measures

#### 2.1.1 Sexual orientation

We assessed sexual orientation using the question, “How do you identify your sexual orientation? … Please choose which one best fits how you identify.” Mutually exclusive response options were: heterosexual/straight, gay/lesbian/homosexual, bisexual, pansexual, queer, asexual, other, unsure/questioning, and “I do not use a label.”

#### 2.1.2 Gender identity

We assessed gender using the question, “What is your current gender? Check all that apply.” Response options were: man/boy, woman/girl, transgender, female to male transgender/FTM, male to female transgender/MTF, trans male/transmasculine, trans female/trans feminine, genderqueer, gender expansive, androgynous, nonbinary, two-spirited, third gender, agender, not sure, and other.

#### 2.1.3 Sex assigned at birth

We measured sex assigned at birth using the question, “What sex were you assigned at birth?” Mutually exclusive response options were: female, male, intersex, other, and “prefer not to say.”

#### 2.1.4 Racial/ethnic identity

We measured racial/ethnic identity using the question, “How do you identify your race/ethnicity? Check all that apply.” Response options were: American Indian or Alaska Native, Asian (including Asian Desi), Black/African American, Hispanic/Latinx, Native Hawaiian or Other Pacific Islander, White/Caucasian (non-Hispanic; includes Middle Eastern), and Other. Participants who indicated more than one response were coded as Multi-racial/Multi-ethnic.

#### 2.1.5 Internalized stigma

Internalized stigma was assessed at pre-intervention, immediately post-intervention, and two-week follow-up using a modified version of the Lesbian, Gay, and Bisexual Identity Scale (LGBIS; Mohr and Kendra, 2011). The LGBIS is a 27-item self-report measure that assesses dimensions of lesbian, gay, and bisexual identity, yielding scores on eight subscales. We used the Internalized Homonegativity subscale, which comprises three items; participants rate their endorsement of these items on a 6-point Likert scale, ranging from 1 (“disagree strongly”) to 6 (“agree strongly”), and an average score is calculated, ranging from 1 to 6, with higher scores indicating greater internalized stigma. For the purposes of this study, items were updated to include additional LGBTQ+ identities. Internal consistency was α = 0.85 at baseline, α = 0.89 at post-intervention, and α = 0.87 at two-week follow-up.

#### 2.1.6 Identity pride

Identity pride was assessed at pre-intervention, immediately post-intervention, and two-week follow-up using a modified version of the LGBIS (see above). Specifically, we used the Identity Affirmation subscale of the LGBIS, which comprises three items; participants rate their endorsement of these items on a 6-point Likert scale, ranging from 1 (“disagree strongly”) to 6 (“agree strongly”), and an average score is calculated from 1 to 6, with higher scores indicating greater identity pride. Internal consistency was α = 0.91 at baseline, α = 0.92 at post-intervention, and α = 0.92 at two-week follow-up.

#### 2.1.7 Hopelessness

Hopelessness was assessed at pre-intervention, immediately post-intervention, and two-week follow-up using the Beck Hopelessness Scale-4 (BHS-4; Perczel Forintos et al., 2013). The BHS-4 is a validated, abbreviated version of the original 20-item measure used to assess hopelessness in youth and has been included as an outcome in previous studies of SSI efficacy (e.g., Schleider et al., 2020, Schleider et al., 2022). Respondents indicate their current level of hopelessness through rating their agreement with four statements (e.g., “My future seems dark to me”) on a 4-point Likert scale (from 0 = “absolutely agree” to 3 = “absolutely agree”). An average score is calculated from 0 to 3, with a higher score indicating greater levels of hopelessness. Internal consistency was α = 0.84 at baseline, α = 0.87 at post-intervention, and α = 0.88 at two-week follow-up.

#### 2.1.8 Depression symptoms

Past-week depression symptoms were assessed at pre-intervention and two-week follow-up using the Children's Depression Inventory, Second Edition: Self-Report Short version (CDI 2:SR[S]; Kovacs, 2015). The CDI 2:SR[S] is a 12-item reliable and valid measure for depression severity in youth which queries a range of depression symptoms, such as irritability and fatigue. Questions are phrased as “Pick the sentence that best describes the way you have been feeling for the past two weeks,” and include a range of depression symptoms, such as irritability and fatigue. Response items for each symptom range in severity on a 3-point Likert scale ranging from 0 (e.g., “I am almost never cranky”), 1 (e.g., “I feel cranky many times”), and 2 (e.g., “I feel cranky all the time”). The CDI 2:SR[S] is measured continuously, and average scores range from 0 to 2, with higher average scores indicating more severe depression symptomatology. Internal consistency was α = 0.83 at baseline and α = 0.84 at follow-up.

#### 2.1.9 Anxiety symptoms

Anxiety symptoms were assessed at pre-intervention using the Generalized Anxiety Disorder – 7 (GAD-7; Spitzer et al., 2006). The GAD-7 comprises 7 items and asks participants to rate how frequently they were bothered by each item over the last two weeks on a Likert scale from 0 (“not at all”) to 3 (“nearly every day”). Response options are on a Likert scale from 0 (“not at all”) to 3 (“nearly every day”). The GAD-7 is measured continuously, and average scores range from 0 to 3; higher average scores reflect greater anxiety symptom severity. Internal consistency was α = 0.87 at baseline and α = 0.89 at follow-up.

#### 2.1.10 Self-hatred

Self-hatred was assessed at pre- and post-intervention using an adapted version of the Self-Hate Scale (SHS; Turnell et al., 2019). The full SHS assesses an individual's level of self-hate over the past year by indicating agreement with seven statements (e.g., “I hate myself”). Our adapted version of the SHS comprised 3 of these 7 items (Schleider et al., 2020). Response options are on a 7-point Likert scale, ranging from 1 (“not at all true for me”) to 7 (“very true for me”). Total scores are calculated using the average of all items, and range from 1 to 3 for this adapted version. The SHS is measured continuously, and higher scores indicate higher levels of self-hate. Internal consistency was α = 0.91 at baseline and α = 0.93 at post-intervention.

#### 2.1.11 Program feedback

All participants were administered the Program Feedback Scale (PFS) at post-intervention (Schleider et al., 2019). The PFS assesses perceived acceptability and feasibility of the SSI by indicating agreement with 7 statements (e.g., “I enjoyed the activity”) on a 5-point Likert scale from 1 (“really disagree”) to 5 (“really agree”). The PFS is measured continuously, and average scores range from 1 to 5; higher average scores reflect greater perceived acceptability.

### 2.2 Statistical analysis

The RStudio Statistical Program was used for analyses (Allaire, 2012). All data and analytic code are available at https://osf.io/kxv4w.

To gauge randomization success, we ran ANOVAs to test condition-based differences in continuous variables (age and pre-randomization levels of identity pride, internalized stigma, hopelessness, self-hatred, depression, and anxiety) and Chi-Square Tests to test condition-based differences in categorical variables (gender, sex assigned at birth, sexual orientation, primary language, race, and grade in school).

To examine the acceptability of Project RISE, we calculated item-level mean post-SSI scores on the Program Feedback Scale. Per previously-employed benchmarks in online SSI trials (Schleider et al., 2022), a pre-registered cutoff of >3.5 on any given program feedback scale item was indicated to reflect endorsement of that item (i.e., positive feedback/adequate acceptability).

To test our main hypotheses (between-group intervention effects), we conducted a series of regressions that examined whether Project RISE, relative to the information-only condition, led to differential changes in identity pride and internalized stigma from baseline to two-week follow up (primary outcomes); hopelessness and depression symptoms from baseline to two-week follow up (secondary outcomes); and identity pride, internalized stigma, hopelessness, and self-hatred from baseline to immediate post-intervention (secondary outcomes).

To examine the robustness of intervention impacts at two-week follow-up, we analyzed and compared regression results using three different approaches to handling missing data. We employed multiple approaches to missing data analysis in order to ensure robustness of results, given the unique strengths and weaknesses inherent to each approach. First, we imputed participant-level missing data using the expectation-maximization and bootstrapping algorithm implemented with Amelia II in R. We imputed as many datasets as there were percentage points of missing data, rounding to the next-highest percentage (for example, if 10.5 % of data was missing, we created 11 imputed datasets), which allowed us to retain high power despite missing data. Next, we ran two sets of completers-only analyses (these were not pre-registered and were included post-hoc to gauge robustness of results). One set of analyses used listwise deletion for participants lacking follow-up data and the other used multiple imputation to estimate follow-up outcomes for all participants, but this latter method excluded those who did not complete their assigned intervention condition (either Project RISE or information-only control).

In addition to between-group effects, we also computed within-group intervention effects via paired *t*-tests (these were added as exploratory, non-pre-registered tests to further characterize each condition's individual-specific impacts on outcomes of interest).

We implemented several best-practices to prevent and exclude fraudulent participants (e.g., bots). These included several layers of external validation (checking for consistent email addresses, IP addresses, and/or false addresses or phone numbers; permitting only one survey per IP address; limiting survey access from non-US IP addresses), embedding CAPTCHAs into surveys (which bots cannot complete); including a screener to ascertain study eligibility prior to allowing participants to consent; employing bot-detection software built into Qualtrics; and excluding participants who provide nonsensical free-text responses within the single-session interventions.

## 3 Results

### 3.1 Sample characteristics

A total of 538 participants were randomized to a condition. Fig. 1 demonstrates the flow of participants in the study. Table 1 includes descriptive statistics for the sample of randomized participants. The average age of participants was 15.06 (*SD* = 0.97). Participants were largely assigned female sex at birth (*N* = 480, 89.22 %). The sample included a high number of participants who identified with a gender different than the one assigned at birth (*N* = 339, 63.01 %). Most participants endorsed an identity of either “Woman/Girl” (*N* = 197, 36.62 %) or “Nonbinary” (*N* = 166, 30.86 %). Participants' sexual orientations included Bisexual (*N* = 131, 24.35 %), Gay/Lesbian/Homosexual (*N* = 130, 24.16 %), Pansexual (*N* = 90, 16.73 %), Queer (*N* = 73, 13.57 %), Asexual (*N* = 38, 7.06 %), Heterosexual/Straight (*N* = 1, 0.2 %), and Other (*N* = 26, 4.83 %; e.g., “Omnisexual”, “Demisexual”). Remaining participants listed that they were unsure or did not use a label (*N* = 49, 9.11 %). Nearly half of participants endorsed a White racial identity (*N* = 259, 48.14 %), with others self-identifying as Multiracial (*N* = 110, 20.45 %), Hispanic/Latinx (*N* = 65, 12.08 %), Black/African American (*N* = 48, 8.92 %), Asian (*N* = 40, 7.43 %), American Indian or Alaska Native (*N* = 6, 1.12 %), Native Hawaiian or other Pacific Islander (*N* = 3, 0.56 %), and Other (*N* = 4, 0.74 %; e.g., “Jewish”, “Middle Eastern”). Three participants preferred not to identify a race/ethnicity (0.56 %). No group differences emerged on demographic factors or baseline psychological outcomes, indicating successful randomization.

**Table 1:** Participant characteristics.

	Project Rise (*N* = 262)	Active control (*N* = 276)
Age	M = 15.05, SD = 0.97	M = 15.07, SD = 0.97
Sex assigned at birth		
Female	235 (89.69 %)	245 (88.77 %)
Male	26 (9.92 %)	30 (10.87 %)
Prefer not to say	1 (0.38 %)	1 (0.36 %)
Different self-identified gender than the sex assigned at birth?		
Yes	157 (59.92 %)	182 (65.94 %)
No	105 (40.08 %)	94 (34.06 %)
Self-identified gender^a^		
Man/boy	49 (18.70 %)	47 (17.03 %)
Woman/girl	100 (38.17 %)	97 (35.14 %)
Transgender	38 (14.50 %)	45 (16.3 %)
Female to male transgender/FTM	31 (11.83 %)	36 (13.04 %)
Male to female transgender/MTF	5 (1.91 %)	8 (2.89 %)
Trans male/transmasculine	45 (17.18 %)	47 (17.03 %)
Trans female/trans feminine	8 (3.05 %)	9 (3.26 %)
Genderqueer	29 (11.07 %)	35 (12.68 %)
Gender expansive	9 (3.44 %)	4 (1.45 %)
Androgynous	29 (11.07 %)	27 (9.78 %)
Nonbinary	81 (30.92 %)	85 (30.79 %)
Two-spirited	2 (0.76 %)	5 (1.81 %)
Third gender	0 (0.0 %)	2 (0.72 %)
Agender	12 (4.58 %)	17 (6.16 %)
Other	37 (14.12 %)	33 (11.96 %)
Not sure	25 (9.54 %)	26 (9.42 %)
Self-identified sexual orientation		
Heterosexual/straight	0 (0.0 %)	1 (0.36 %)
Gay/lesbian/homosexual	61 (23.28 %)	69 (25 %)
Bisexual	61 (23.28 %)	70 (25.36 %)
Pansexual	42 (16.03 %)	48 (17.39 %)
Queer	35 (13.36 %)	38 (13.77 %)
Asexual	20 (7.63 %)	18 (6.52 %)
Unsure/questioning	19 (7.25 %)	8 (2.89 %)
Other	11 (4.19 %)	15 (5.43 %)
Do not use label	13 (4.96 %)	9 (3.26 %)
Race		
American Indian or Alaska Native	3 (1.15 %)	3 (1.09 %)
Asian	19 (7.25 %)	21 (7.61 %)
Black/African American	27 (10.31 %)	21 (7.61 %)
Hispanic/Latinx	29 (11.07 %)	36 (13.04 %)
Native Hawaiian or other Pacific Islander	2 (0.76 %)	1 (0.36 %)
White/Caucasian	127 (48.47 %)	132 (47.83 %)
Multiracial	53 (20.23 %)	57 (20.65 %)
Other race	1 (0.38 %)	3 (1.09 %)
Prefer not to answer	1 (0.38 %)	2 (0.72 %)
Primary language		
English	253 (96.56 %)	265 (96.01 %)
Chinese	0 (0.0 %)	1 (0.36 %)
French	1 (0.38 %)	1 (0.36 %)
Spanish	4 (1.53 %)	6 (2.17 %)
Other	4 (1.53 %)	3 (1.09 %)
Grade		
7th grade	9 (3.44 %)	10 (3.62 %)
8th grade	34 (12.98 %)	38 (13.77 %)
9th grade	75 (28.63 %)	73 (26.45 %)
10th grade	92 (35.11 %)	104 (37.68 %)
11th grade	49 (18.70 %)	49 (17.75 %)
12th grade	3 (1.15 %)	2 (0.72 %)
Negative impact that LGBT stigma has had on life	M = 6.33, SD = 2.28	M = 6.04, SD = 2.34

a Participants were permitted to choose multiple genders.

### 3.2 Intervention completion rates and acceptability

Among adolescents randomized to Project RISE, 87.02 % (*N* = 228) fully completed the intervention, 8.78 % (*N* = 23) dropped-out prior to completing the psychoeducation, 3.05 % (*N* = 8) completed psychoeducation but dropped-out prior to completing a personalized coping plan, and 1.15 % (*N* = 3) completed a personalized coping plan but dropped-out before finishing the intervention in full. Both participants who completed the intervention and those who completed the active control each found their assigned condition acceptable across all Program Feedback Scale (PFS) items, as indicated by the item-level means being above 3.5/5 for both conditions (Table 2).

**Table 2:** Program Feedback Scale results.

PFS items (range across items, 1–5)	Project Rise (*N* = 225)		Active control (*N* = 269)	
	Mean	SD	Mean	SD
Enjoyed	4.09	0.65	3.74	0.67
Understood	4.45	0.57	4.38	0.61
Easy to use	4.44	0.64	4.43	0.63
Tried my hardest	4.53	0.63	4.43	0.68
Helpful to other kids	4.52	0.62	4.22	0.71
Would recommend to a friend	4.17	0.85	3.67	0.99
Agree with message	4.68	0.55	4.49	0.58

### 3.3 Differential SSI and follow-up survey completion

Of the participants who were randomly assigned to an experimental condition, 59.85 % fully completed the baseline outcomes and the two-week follow-up survey. Participants in Project RISE completed the follow-up at a significantly lower rate (χ2(1) = 3.96, *p* = 0.04; 55.34 %) than participants in the information-only control (64.13 %), potentially because participants in the comparison group were told they would receive access Project RISE after the completion of the follow-up assessment (though we could not test this prospect directly).

### 3.4 Internalized stigma outcomes

Tables 3 (full sample) and 4 (intervention completers-only) present means and standard deviations for all program outcomes by experimental condition at baseline, post-intervention, and two-week follow-up. Participants in Project RISE reported greater decreases in internalized stigma from baseline to immediately post-intervention compared to participants in the control condition (*t*(475) = −5.37, *p* < 0.001, *d* = −0.49; 95 % CI: −0.68, −0.31). Using listwise deletion data from completers-only, participants in Project RISE similarly reported greater decreases in internalized stigma from baseline to two-week follow-up compared to participants in the control condition (*t*(332) = −2.38, *p* = 0.018, *d* = −0.26; 95 % CI: −0.48, −0.05). Analyses using multiple imputation for missing data from intervention completers-only suggested similar findings as did the other two missingness approaches (*t*(332) = −2.73, *p* = 0.006, *d* = −0.30; 95 % CI: −0.52, −0.08). Analyses using multiple imputation for missing data from the entire sample (including individuals who did not finish the intervention) also suggested similar findings (*t*(339) = −2.12, *p* = 0.03, *d* = −0.23; 95 % CI: −0.44, −0.02).

**Table 3:** Means and standard deviations for the full sample.

	Active control			Project Rise		
	N	Mean	SD	N	Mean	SD
Identity pride						
Baseline	274	5.06	0.92	262	5.04	1.01
Immediate-post	257	5.08	0.99	219	5.21	0.94
2-week follow-up	185	5.03	0.95	155	4.97	1.04
Internalized stigma						
Baseline	276	2.74	1.30	262	2.77	1.30
Immediate-post	257	2.70	1.33	219	2.42	1.27
2-week follow-up	185	2.65	1.32	155	2.60	1.27
Hopelessness						
Baseline	276	1.53	0.74	262	1.52	0.73
Immediate-post	251	1.38	0.76	216	1.23	0.72
2-week follow-up	183	1.28	0.73	155	1.42	0.79
Self-hatred						
Baseline	276	4.25	1.75	261	4.30	1.77
Immediate-post	265	3.96	1.85	223	3.65	1.73
Depression						
Baseline	276	1.05	0.38	262	1.10	0.38
2-week follow-up	182	0.92	0.40	152	0.96	0.39
Anxiety						
Baseline	276	1.74	0.75	262	1.74	0.73
2-week follow-up	182	1.52	0.79	152	1.62	0.76

Regarding within-group effects, participants assigned to Project RISE reported significant reductions in internalized stigma immediately after the intervention (*t*(218) = −7.39 *p* < 0.001; *d* = −0.71; 95 % CI: −0.9, −0.51) and at two-week follow-up (*t*(147) = −5.13; *p* < 0.001; *d* = −0.60; 95 % CI: −0.83, −0.36). Conversely, active control-group participants did not report significant reductions in internalized stigma immediately after the intervention (*t*(256) = −1.67; *p* = 0.09; *d* = −0.15; 95 % CI, −0.32, 0.03) or at two-week follow-up (*t*(184) = −0.71; *p* = 0.48; *d* = −0.07; 95 % CI, −0.28, 0.13).

### 3.5 Identity pride outcomes

Participants in Project RISE reported greater increases in Identity Pride from baseline to immediately post-intervention compared to participants in the control condition (*t*(475) = 2.73, *p* = 0.007, *d* = 0.25; 95 % CI: 0.07, 0.43). Using listwise deletion data from completers-only, there did not appear to be a significant effect of condition on Identity Pride scores at two-week follow up (*t*(332) = 1.41*, p* = 0.159, *d* = 0.16; 95 % CI: −0.06, 0.37). Analyses using multiple imputation for missing data from completers-only suggested similar findings as to analyses using listwise deletion (*t*(332) = 1.36, *p* = 0.173, *d* = 0.15; 95 % CI: −0.07, 0.37). Analyses using multiple imputation for missing data from the entire sample (including individuals who did not finish the intervention) also suggested similar findings (*t*(475) = 1.39, *p* = 0.163, *d* = 0.13; 95 % CI: −0.05, 0.31).

Regarding within-group effects, there was a statistically significant within-group increase in identity pride among participants in the Project RISE condition immediately after the intervention (*t*(218) = 4.46; *p* < 0.001; *d* = 0.43; 95 % CI, 0.24, 0.62) and at two-week follow-up (*t*(147) = 2.02; *p* = 0.04; *d* = 0.24; 95 % CI: 0.01, 0.46). There was no significant within-group change in identity pride among participants in the active control condition immediately after the intervention (*t*(256) = 1.49; *p* = 0.14; *d* = 0.13; 95 % CI: −0.04, 0.3) or at two-week follow-up (*t*(184) = −0.62; *p* = 0.53; *d* = −0.06; 95 % CI: −0.27, 0.14) (Table 4).

**Table 4:** Means and standard deviations for completers only.

	Active control			Project Rise		
	N	Mean	SD	N	Mean	SD
Identity pride						
Baseline	185	5.06	0.86	148	4.87	1.11
Immediate-post	185	5.11	0.91	148	5.11	1.03
2-week follow-up	185	5.03	0.95	148	4.97	1.04
Internalized stigma						
Baseline	185	2.70	1.27	148	2.89	1.36
Immediate-post	185	2.63	1.27	148	2.47	1.31
2-week follow-up	185	2.65	1.32	148	2.58	1.26
Hopelessness						
Baseline	183	1.47	0.76	148	1.49	0.74
Immediate-post	183	1.33	0.75	148	1.20	0.73
2-week follow-up	183	1.28	0.73	148	1.42	0.79
Self-hatred						
Baseline	265	4.22	1.76	223	4.30	1.76
Immediate-post	265	3.96	1.85	223	3.65	1.73
Depression						
Baseline	180	1.02	0.76	143	1.10	0.38
2-week follow-up	180	0.92	0.79	143	0.96	0.39
Anxiety						
Baseline	182	1.66	0.76	145	1.79	0.71
2-week follow-up	182	1.52	0.79	145	1.62	0.75

### 3.6 Hopelessness, self-hate, depression, and anxiety outcomes

Participants in Project RISE reported greater decreases in hopelessness from baseline to immediately post-intervention compared to participants in the control condition (*t*(466) = −3.92, *p* < 0.001, *d* = −0.36; 95 % CI: −0.55, −0.18). Using listwise deletion data from completers-only, participants in Project RISE reported smaller reductions in hopelessness scores at follow up compared to participants in the control condition (*t*(330) = 1.99, *p* = 0.046, *d* = 0.22; 95 % CI: 0.004, 0.44). However, in analyses using multiple imputation for missing data from completers-only, condition showed no effect on changes in hopelessness scores from baseline to follow-up (*t*(330) = 01.75, *p* = 0.08, *d* = 0.19; 95 % CI: −0.02, 0.41), and within-group effects (described below) suggested numerical decreases in hopelessness scores from baseline to two-week follow-up in both conditions. Analyses using multiple imputation for missing data from the entire sample (including individuals who did not finish the intervention) also suggested no differences in two-week hopelessness outcomes by condition (*t*(337) = 1.74, *p* = 0.08, *d* = 0.19; 95 % CI: −0.03, 0.40).

Regarding within-group effects, there was a statistically significant within-group reduction in hopelessness among participants in the Project RISE condition immediately after the intervention (*t*(215) = −7.98, *p* < 0.001; *d* = −0.77; 95 % CI: −0.96, −0.57) and no significant change in hopelessness from baseline to two-week follow-up (*t*(147) = −1.31; *p* = 0.19; *d* = −0.15; 95 % CI: −0.38, 0.08). There was a statistically significant within-group reduction in hopelessness among participants in the active control condition immediately after the intervention (*t*(250) = −4.77; *p* < 0.001; *d* = −0.43; 95 % CI: −0.60, −0.25) and at two-week follow-up (*t*(182) = −4.29; *p* < 0.001; *d* = −0.45; 95 % CI: −0.66, −0.24).

Using listwise deletion data from completers-only, changes in depression symptoms did not differ by condition from baseline to two-week follow-up (*t*(322) = −0.78, *p* = 0.43, *d* = −0.09; 95 % CI: −0.31, 0.13). Analyses using multiple imputation for missing data from completers-only suggested similar findings (*t*(322) = −0.93, *p* = 0.35, *d* = −0.10; 95 % CI, −0.32, 0.12). Analyses using multiple imputation for missing data from the entire sample (including individuals who did not finish the intervention) also suggested similar findings (*t*(333) = −1.03, *p* = 0.30, *d* = −0.11; 95 % CI: −0.33, 0.10). However, within-group effects suggested decreases in depression for participants in *both* conditions. There were statistically significant within-group reductions in depression among participants in the Project RISE condition (*t*(142) = −5.96; *p* < 0.001; *d* = −0.68; 95 % CI: −0.92, −0.44) and in the active control condition (*t*(179) = −5.07, *p* < 0.001; *d* = −0.53; 95 % CI: −0.74, −0.32) at two-week follow-up.

Using listwise deletion data from completers-only, there did not appear to be a significant effect of condition on anxiety scores at follow up (*t*(326) = 0.12, *p* = 0.91, *d* = 0.01; 95 % CI: −0.20, 0.23). Analyses using multiple imputation for missing data from completers-only suggested similar findings (*t*(326) = 0.13, *p* = 0.09, *d* = 0.01; 95 % CI: −0.20, 0.23). Analyses using multiple imputation for missing data from the entire sample (including individuals who did not finish the intervention) also suggested similar findings (*t*(333) = 0.01, *p* = 0.09, *d* = 0.001; 95 % CI: −0.21, 0.22). However, within-group effects suggested decreases in anxiety for participants in *both* experimental conditions. There were statistically significant within-group reductions in anxiety among participants in the Project RISE condition (*t*(144) = −3.24; *p* < 0.001; *d* = −0.38; 95 % CI: −0.61, −0.15) and in the active control condition (*t*(182) = −3.22, *p* < 0.001; *d* = −0.34; 95 % CI: −0.54, −0.13) at two-week follow-up.

Participants in Project RISE reported greater decreases in self-hate from baseline to immediately post-intervention compared to participants in the control condition (*t*(487) = −5.27, *p* < 0.001, *d* = −0.48; 95 % CI: −0.66, −0.29). There was a statistically significant within-group reduction in self-hatred among participants in the Project RISE condition (*t*(221) = −10.09; *p* < 0.001; *d* = −0.96; 95 % CI: −1.15, −0.76) and participants in the active control condition (*t*(264) = −6.47; *p* < 0.001; *d* = −0.56; 95 % CI: −0.74, −0.39) from baseline to immediately post-intervention.

## 4 Discussion

We evaluated a novel online SSI (‘Project RISE’) designed to combat minority stress by reducing internalized stigma in LGBTQ+ young people. Overall, RISE successfully improved post-intervention (internalized stigma, identity pride, hopelessness) and two-week (internalized stigma) outcomes, relative to a psychoeducational control. RISE was also acceptable to, and completed at a high rate (89 %) by, participants. RISE did not outperform the control in reducing secondary outcomes of interest at 2-week follow-up (hopelessness, self-hate, depression, and anxiety symptoms); instead, levels of secondary 2-week outcomes tended to improve in participants regardless of condition assignment. Overall, results suggest the promise of Project RISE to help mitigate internalized stigma in LGBTQ+ young people, at least in the short-term.

Project RISE outperformed the control condition across all outcomes at immediate post-intervention and was rated as highly acceptable. At two-week follow-up, in both sets of analyses using completers-only data (but not in full-sample analyses, which included intervention non-completers), youth assigned to Project RISE reported greater decreases in internalized stigma from baseline to two-week follow up. Between-group differences were attenuated at two-week follow-up for other outcomes, with no significant effects of condition (i.e., RISE vs. information-only control) on identity pride, self-hatred, depression, or anxiety symptoms. However, within-group effect sizes suggested significant improvements in identity pride, depression, anxiety, and self-hate across *both* conditions at two-week follow-up. The discrepancy between these non-significant between-group effects and these significant within-group effects across conditions may reflect the strength of our control condition (which included psychoeducation on minority stress theory). Notably, using listwise deletion data from completers-only, participants in Project RISE reported greater hopelessness scores at two-week follow-up compared to participants in the control condition, but this effect did not hold across the two other missing data approaches, and within-group effects suggested numerical reductions in hopelessness across both conditions. Reductions in hopelessness in this study were numerically larger than those observed in a recent study involving a high-symptom youth sample and non-internalized stigma focused online SSIs (d = 0.16–0.28; Schleider et al., 2022). While these findings do not necessarily index clinical significance, it is notable that improvements in hopelessness in this study were greater than for other SSIs with documented efficacy in reducing adolescent depression symptoms.

Although the differences in outcomes between intention-to-treat and completers-only analyses were unexpected, our findings highlight the strength of Project RISE: participants who completed the intervention tended to benefit from it. Additionally, our findings also underscore potential processes related to attrition; namely, fewer participants who received the RISE than who received the control program completed the follow-up. This difference may be because these participants already received the intervention, and thus the immediate intervention-derived benefits may have reduced the perceived need to complete the follow-up. Conversely, participants in the control condition, who had not yet received the intervention, may have been more likely to complete the follow-up survey, as they were aware that the follow-up would include new resources and forms of support. Regardless, future research may examine ways to boost engagement with Project RISE among youths who access it, given benefits observed for full-completers. On the other hand, it is likely that individuals who only completed a portion of the SSI received an intervention that equated to higher similarity to the control condition, which comprised psychoeducation on minority stress, relative to those who completed the RISE SSI in full.

Findings suggest that Project RISE may have utility in reducing internalized consequences of minority stress and increasing identity pride among LGBTQ+ young people. By mitigating many barriers that traditionally preclude LGBTQ+ young people from accessing mental health interventions, online SSIs have the potential to rapidly expand access to services for this population. Moreover, interventions providing psychoeducation on minority stress processes may help reduce the severity of minority stress consequences for LGBTQ+ young people.

### 4.1 Immediate versus two-week outcomes

Overall, analyses revealed within-group improvements across all outcome variables, both for participants who completed Project RISE and for those who completed the control condition. Both conditions were rated as highly acceptable by participants. Notably, both conditions included psychoeducation regarding minority stress theory, given the aforementioned ethical importance of sharing this information with participants and the methodological importance of using a strong control condition to test our intervention. These findings thus suggest that learning about minority stress theory, in and of itself, may be beneficial for LGBTQ+ young people.

When outcomes were assessed immediately post-intervention, Project RISE outperformed the control condition on all measures. Some of these initial differences in outcomes may have been due to the interactivity of RISE (e.g., activities soliciting participant engagement and encouraging participants to tie content to examples from their own lives), compared to the more passive nature of the information-only control (although we could not test this possibility directly). However, at two-week follow-up, we found no significant differences by condition on measures of identity pride, self-hatred, or depression.

Why might Project RISE's effects on *internalized stigma*, in particular, have persisted at two-week follow-up relative to the information-only control? One interpretation of these findings is that RISE targeted internalized stigma more directly than did the control condition. Education on the effects of minority stress may have challenged longer-term self-stigmatizing cognitions among participants who completed Project RISE by teaching participants that adverse consequences stemming from minority stress experiences are *not* their fault, but *are* to some extent within their power to mitigate (i.e., restoring a degree of agency, and doing so through narratives, which have been shown to be an impactful means of messaging, Schleider et al., 2020). Given this study's design as a randomized controlled trial with a robust control condition, the fact that differences still emerged speaks to the strength of the intervention and the efficacy of psychoeducation on minority stress processes for LGBTQ+ young people.

### 4.2 Importance of waiving parental permission

Adolescents self-referred into the SSI. There is existing legislation across many states in the US that enable adolescents aged ≥12 to consent to mental healthcare without a parent or guardian, such as to increase the likelihood of seeking and obtaining care among adolescents. Allowing adolescents to participate in minimal-risk online SSIs without requiring parental consent may be critical in increasing treatment access and use. This study demonstrates that adolescents can safely participate in online SSIs and that SSIs may improve both short-term and longer-term mental health-related outcomes. Requiring parent or guardian approval for adolescents to try online interventions could prevent thousands of youth from receiving minimal-risk, free, and evidence-based support (Samargia et al., 2006; Wilson and Deane, 2012) and may disproportionately impact LGBTQ+ youth and youth of color, for whom concerns about caregiver stigma or dismissal (e.g., as related to the rejection of one's identity, beliefs about seeking mental health treatment, or both) often prevent youth from disclosing treatment needs to family (Brown et al., 2016). The SSI tested in this study posed minimal risk to safety and may offer an effective path to reducing the impact of minority stress and increasing access to mental health support for adolescents.

### 4.3 Limitations and future directions

Results should be considered in context of this study's limitations. Participation in the study relied on adolescents having the interest and time to complete the study, their comfort with English, and their Internet access. Additionally, adolescents assigned female at birth represented the majority of the sample. Our findings thus may not apply uniformly across adolescents. However, previous trials of psychosocial interventions for adolescents have largely required parent or guardian consent; by eliminating this barrier, this study may have included adolescents underrepresented in such prior work, including adolescents who are not open with caregivers about mental health difficulties or LGBTQ+ identities. Moreover, 273 adolescents in our recruitment sample completed the assent but did not complete the baseline survey and were thus not randomized to a condition, which may reflect sampling biases. In addition, given the apparent purpose of the intervention (i.e., mitigating effects of LGBTQ+ minority stress), it is possible that treatment effects may reflect study demand characteristics, where adolescents may have overreported positive outcomes and underreported negative outcomes due to being aware of the study's purpose. Further, we did not formally evaluate adolescents' use of other mental health supports during the study period. Some research suggests external mental health supports may not impact SSI utility: for instance, a previous randomized trial including 96 high-symptom adolescents demonstrated no effect of external mental health treatment (e.g., psychotherapy and/or medication) on SSI response across nine-month follow-up (Schleider and Weisz, 2018). Future trials may consider assessing whether benefits of SSIs for LGBTQ+ youth shift in the context of other mental health supports they use. Finally, this study employed a relatively brief follow-up period (i.e., two weeks). This study was intended as a pilot study to examine initial efficacy of the tested SSI; thus, we were interested in whether this SSI could modify proximal, short-term mechanisms underlying mental health trajectories. Future work will include a larger-scale examination of the efficacy of this intervention, using a longer follow-up period, to examine changes in outcomes over a longer period of time.

### 4.4 Strengths

Our study has some notable strengths. In particular, results demonstrate the utility of an easily accessible intervention targeting the effects of minority stress for LGBTQ+ youth, which is critically needed in light of the minority stressors and resultant health disparities this population faces, and particularly so for transgender youth. Additionally, the online nature of our intervention increases its accessibility for LGBTQ+ youth, a population which literature suggests may particularly benefit from online delivery of mental health supports (Perry et al., 2018; Schrager et al., 2019). Finally, throughout our study, we incorporated methodologically rigorous and open-access strategies, including pre-registration of our study design and open access to our data from this study.

## 5 Conclusions

Our study demonstrates that Project RISE, a novel online SSI, immediately decreases internalized stigma, hopelessness, self-hatred, and depression; significantly increases identity pride in LGBTQ+ adolescents; and yields sustained reductions in internalized stigma two weeks later. Overall, results indicate that future interventions should incorporate developmentally-appropriate psychoeducation on the impacts of minority stress in conjunction with engaging activities to enhance participant learning. Future work may assess this SSI among adolescents using other languages and those who may not have stable Internet access, and other work may examine the implementation of this SSI in conjunction with other mental health supports for LGBTQ+ youth.

## Declaration of competing interest

JLS serves on the Scientific Advisory Board for Walden Wise and the Clinical Advisory Board for Koko; is Co-Founder and Co-Director of Single Session Support Solutions. Inc.; and receives book royalties from New Harbinger, Oxford University Press, and Little Brown Book Group.

## Appendix A Supplementary data

Supplementary figures Image 1

## Footnotes

3 Because the primary outcome in this study (internalized stigma) was not health-related, and because we were not focused on a high-symptom sample of youth, we did not register this study as a “clinical trial” on clinicaltrials.gov. All methods were pre-registered on OSF prior to enrollment of the first participant in the study.

## References

[bb0005] Allaire J. (2012). RStudio: integrated development environment for R.

[bb0010] Brooks V.R. (1981).

[bb0015] Brown A., Rice S.M., Rickwood D.J., Parker A.G. (2016). Systematic review of barriers and facilitators to accessing and engaging with mental health care among at-risk young people. Asia Pac. Psychiatry.

[bb0020] Chaudoir S.R., Wang K., Pachankis J.E. (2017). What reduces sexual minority stress? A review of the intervention “toolkit”. J. Soc. Issues.

[bb0025] Chodzen G., Hidalgo M.A., Chen D., Garofalo R. (2019). Minority stress factors associated with depression and anxiety among transgender and gender-nonconforming youth. J. Adolesc. Health.

[bb0030] Delozier A.M., Kamody R.C., Rodgers S., Chen D. (2020). Health disparities in transgender and gender expansive adolescents: a topical review from a minority stress framework. J. Pediatr. Psychol..

[bb0035] Fulginiti A., Rhoades H., Mamey M.R., Klemmer C., Srivastava A., Weskamp G., Goldbach J.T. (2021). Sexual minority stress, mental health symptoms, and suicidality among LGBTQ youth accessing crisis services. J. Youth Adolesc..

[bb0040] Guz S., Kattari S.K., Atteberry-Ash B., Klemmer C.L., Call J., Kattari L. (2021). Depression and suicide risk at the cross-section of sexual orientation and gender identity for youth. J. Adolesc. Health.

[bb0045] Hatzenbuehler M.L. (2009). How does sexual minority stigma “get under the skin”? A psychological mediation framework. Psychol. Bull..

[bb0050] Israel T., Choi A.Y., Goodman J.A., Matsuno E., Lin Y.-J., Kary K.G., Merrill C.R.S. (2019). Reducing internalized binegativity: development and efficacy of an online intervention. Psychol. Sex. Orientat. Gend. Divers..

[bb0055] Israel T., Goodman J.A., Kary K.G., Matsuno E., Choi A.Y., Lin Y.-J., Merrill C.R.S. (2021). Development and efficacy of an online intervention targeting lesbian internalized homonegativity. Prof. Psychol. Res. Pract..

[bb0060] Israel T., Matsuno E., Choi A.Y., Goodman J.A., Lin Y.-J., Kary K.G., Merrill C.R.S. (2021). Reducing internalized transnegativity: randomized controlled trial of an online intervention. Psychol. Sex. Orientat. Gend. Divers..

[bb0065] Kovacs M. (2015). The Encyclopedia of Clinical Psychology.

[bb0070] Layland E.K., Carter J.A., Perry N.S., Cienfuegos-Szalay J., Nelson K.M., Bonner C.P., Rendina H.J. (2020). A systematic review of stigma in sexual and gender minority health interventions. Transl. Behav. Med..

[bb0075] Lin Y.-J., Israel T. (2012). A computer-based intervention to reduce internalized heterosexism in men. J. Couns. Psychol..

[bb0080] Mandel S.H. (2014). Targeting sexual stigma: the hybrid case study of “Adam”. Pragmat. Case Stud. Psychother..

[bb0085] Marshal M.P., Dietz L.J., Friedman M.S., Stall R., Smith H.A., McGinley J., Thoma B.C., Murray P.J., D’Augelli A.R., Brent D.A. (2011). Suicidality and depression disparities between sexual minority and heterosexual youth: a meta-analytic review. J. Adolesc. Health.

[bb0090] McDanal R., Rubin A., Fox K.R., Schleider J.L. (2022). Associations of LGBTQ+ identities with acceptability and efficacy of online single-session youth mental health interventions. Behav. Ther..

[bb0095] Meyer I. (1995). Minority stress and mental health in gay men. J. Health Soc. Behav..

[bb0100] Mohr J.J., Kendra M.S. (2011). Revision and extension of a multidimensional measure of sexual minority identity: the Lesbian, Gay, and Bisexual Identity Scale. J. Couns. Psychol..

[bb0105] Nappa M.R., Bartolo M.G., Pistella J., Petrocchi N., Costabile A., Baiocco R. (2022). “I do not like being me”: the impact of self-hate on increased risky sexual behavior in sexual minority people. Sex. Res. Soc. Policy.

[bb0110] Pachankis J.E., Williams S.L., Behari K., Job S., McConocha E.M., Chaudoir S.R. (2020). Brief online interventions for LGBTQ young adult mental and behavioral health: a randomized controlled trial in a high-stigma, low-resource context. J. Consult. Clin. Psychol..

[bb0115] Pachankis J.E., Soulliard Z.A., Morris F., Seager van Dyk I. (2022). A model for adapting evidence-based interventions to be LGBQ-affirmative: putting minority stress principles and case conceptualization into clinical research and practice. Cogn. Behav. Pract..

[bb0120] Perczel Forintos D., Rózsa S., Pilling J., Kopp M. (2013). Proposal for a short version of the Beck Hopelessness Scale based on a national representative survey in Hungary. Community Ment. Health J..

[bb0125] Perry Y., Strauss P., Lin A. (2018). Online interventions for the mental health needs of trans and gender diverse young people. Lancet Psychiatry.

[bb0130] Plöderl M., Tremblay P. (2015). Mental health of sexual minorities. A systematic review. Int. Rev. Psychiatry.

[bb0135] Proujansky R.A., Pachankis J.E. (2014). Toward formulating evidence-based principles of LGB-affirmative psychotherapy. Pragmat. Case Stud. Psychother..

[bb0140] Roe S. (2017). “Family support would have been like amazing”: LGBTQ youth experiences with parental and family support. Fam. J..

[bb0145] Salerno J.P., Williams N.D., Gattamorta K.A. (2020). LGBTQ populations: psychologically vulnerable communities in the COVID-19 pandemic. Psychol. Trauma Theory Res. Pract. Policy.

[bb0150] Samargia L.A., Saewyc E.M., Elliott B.A. (2006). Foregone mental health care and self-reported access barriers among adolescents. J. School Nurs..

[bb0155] Schleider J.L., Weisz J. (2018). A single-session growth mindset intervention for adolescent anxiety and depression: 9-month outcomes of a randomized trial. J. Child Psychol. Psychiatry Allied Discip..

[bb9000] Schleider J.L., Mullarkey M.C., Weisz J.R. (2019). Virtual Reality and Web-Based Growth Mindset Interventions for Adolescent Depression: Protocol for a Three-Arm Randomized Trial. JMIR Res. Protocol..

[bb0160] Schleider J.L., Dobias M.L., Sung J.Y., Mullarkey M.C. (2020). Future directions in single-session youth mental health interventions. J. Clin. Child Adolesc. Psychol..

[bb0165] Schleider J.L., Mullarkey M.C., Fox K.R., Dobias M.L., Shroff A., Hart E.A., Roulston C.A. (2022). A randomized trial of online single-session interventions for adolescent depression during COVID-19. Nat. Hum. Behav..

[bb0170] Schrager S.M., Steiner R.J., Bouris A.M., Macapagal K., Brown C.H. (2019). Methodological considerations for advancing research on the health and wellbeing of sexual and gender minority youth. LGBT Health.

[bb0175] Spitzer R.L., Kroenke K., Williams J.B.W., Löwe B. (2006). A brief measure for assessing generalized anxiety disorder: the GAD-7. Arch. Intern. Med..

[bb0180] Turnell A.I., Fassnacht D.B., Batterham P.J., Calear A.L., Kyrios M. (2019). The Self-Hate Scale: development and validation of a brief measure and its relationship to suicidal ideation. J. Affect. Disord..

[bb0185] Wilson C.J., Deane F.P. (2012). Brief report: need for autonomy and other perceived barriers relating to adolescents’ intentions to seek professional mental health care. J. Adolesc..

