# Supplementary material for: Randomized evaluation of an online single-session intervention for minority stress in LGBTQ+ adolescents
Source: Internet Interv. 2023 Jun 7;33:100633. doi: 10.1016/j.invent.2023.100633 (PMC10457524; doi:10.1016/j.invent.2023.100633)

Online Supplement

**Randomized Evaluation of an Online Single-Session Intervention for Minority Stress in LGBTQ+ Adolescents**

Shen, J.^1^ *, Rubin, A.^2,^*, Cohen, K.^1^, Hart, E. A.^2^, Sung, J.^1^, McDanal, R.^1^, Roulston, C.^1^, Sotomayor, I.^1^, Fox, K. R. ^2^ **, Schleider, J. L. ^1^ **

*denotes equal first-authorship

******denotes equal senior-authorship

^1^ Department of Psychology, Stony Brook University

^2^ Department of Psychology, University of Denver

**Corresponding author**: Jessica L. Schleider, Ph.D. ([jessica.schleider@stonybrook.edu](mailto:jessica.schleider@stonybrook.edu))

**Contents:**

Supplementary Figure 1. Instagram Recruitment Advertisement, and Supplementary Figure 2. Action Card Template from Intervention

Supplementary Figure 1. Instagram Recruitment Advertisement


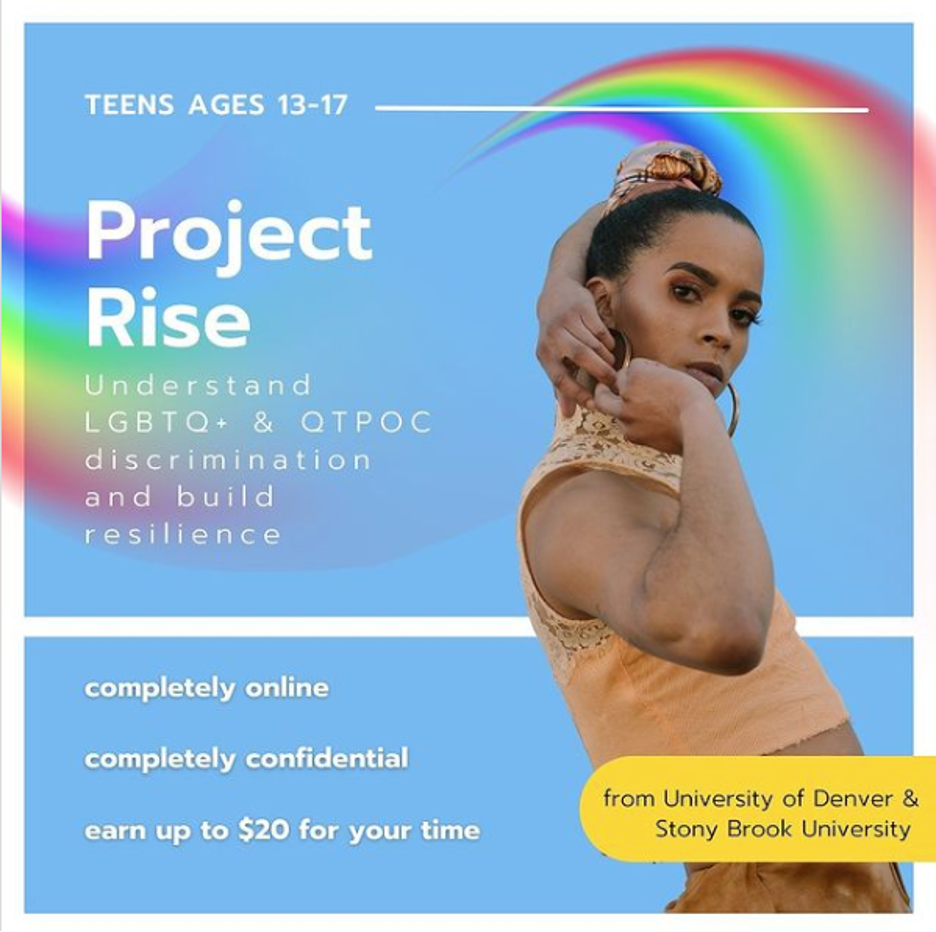


Supplementary Figure 2. Action Card Template from Intervention
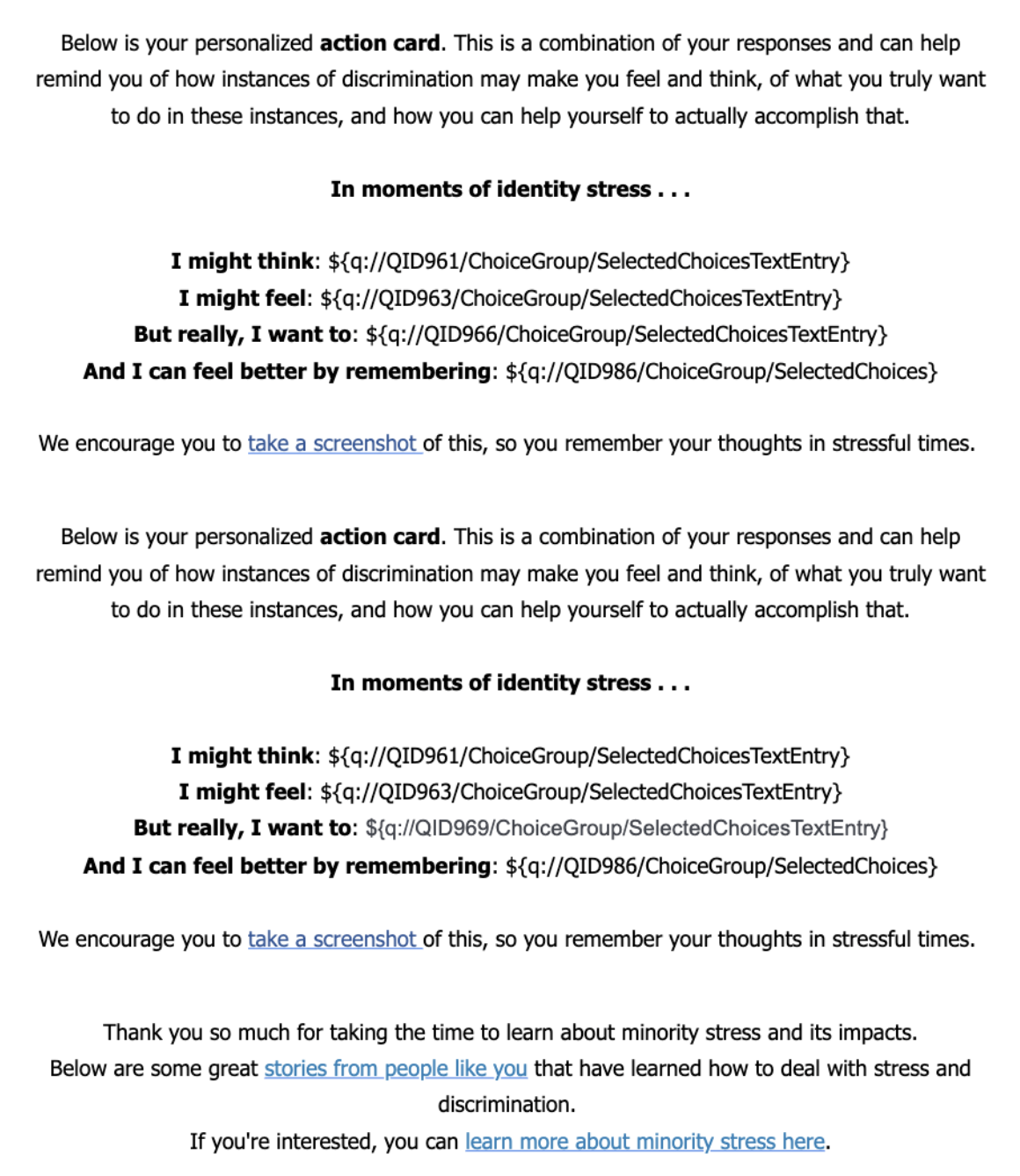

Supplement: Supplementary file 1 — Supplementary figures [file mmc1.docx]
